# Erdafitinib Antagonizes ABCB1-Mediated Multidrug Resistance in Cancer Cells

**DOI:** 10.3389/fonc.2020.00955

**Published:** 2020-06-25

**Authors:** Weiguo Feng, Meng Zhang, Zhuo-Xun Wu, Jing-Quan Wang, Xing-Duo Dong, Yuqi Yang, Qiu-Xu Teng, Xuan-Yu Chen, Qingbin Cui, Dong-Hua Yang

**Affiliations:** ^1^College of Bioscience and Technology, Weifang Medical University, Weifang, China; ^2^Department of Pharmaceutical Sciences, College of Pharmacy and Health Sciences, St. John's University, Queens, NY, United States; ^3^First Clinical Medical College, Shandong University of Traditional Chinese Medicine, Jinan, China; ^4^College of Integrated Chinese and Western Medicine, Hebei Medical University, Shijiazhuang, China

**Keywords:** erdafitinib, ABCB1, multidrug resistance, reversal effect, cancer

## Abstract

ABCB1 overexpression is known to contribute to multidrug resistance (MDR) in cancers. Therefore, it is critical to find effective drugs to target ABCB1 and overcome MDR. Erdafitinib is a tyrosine kinase inhibitor (TKI) of fibroblast growth factor receptor (FGFR) that is approved by the FDA to treat urothelial carcinoma. Previous studies have demonstrated that some TKIs exhibit MDR reversal effect. In this work, we examined whether erdafitinib could reverse MDR mediated by ABCB1. The results of reversal experiments showed that erdafitinib remarkably reversed ABCB1-mediated MDR without affecting ABCG2-mediated MDR. The results of immunofluorescence and Western blot analysis demonstrated that erdafitinib did not affect the expression of ABCB1 or its cellular localization. Further study revealed that erdafitinib inhibited ABCB1 efflux function leading to increasing intracellular drug accumulation, thereby reversing MDR. Furthermore, ATPase assay indicated that erdafitinib activated the ABCB1 ATPase activity. Docking study suggested that erdafitinib interacted with ABCB1 on the drug-binding sites. In summary, this study demonstrated that erdafitinib can reverse MDR mediated by ABCB1, suggesting that combination of erdafitinib and ABCB1-substrate conventional chemotherapeutic drugs could potentially be used to overcome MDR mediated by ABCB1.

## Introduction

Multidrug resistance (MDR) is one of the main reasons leading to failure of chemotherapy in cancers (1, 2). Several mechanisms of MDR have been elucidated, including the increase of intracellular drug efflux (3), cell apoptosis inhibition (4), DNA repair enhancement (5) oncogene mutations (6) and others. Among them, ATP-binding cassette (ABC) transporters-mediated drug resistance is one of the most important factor causing MDR in cancer (7).

ABC transporters are a superfamily which consists of seven subfamilies from A to G, located on cell membranes to execute the pharmacological and physiological functions (8). Among them, ABCB1 (P-gp, MDR1) and ABCG2 (breast cancer resistance protein, BCRP) have been identified as the major contributor to MDR in variety of cancers (3, 9–11). Many studies suggested that the overexpression of ABCB1 and ABCG2 cause reduction of intracellular drugs (12–18). The well-known substrates for ABCB1 transporters are doxorubicin, paclitaxel, colchicine, and vincristine et al. (19). ABCG2 transporter substrates include doxorubicin, mitoxantrone, SN-38, and topotecan et al. (20). Currently, there is no single drug which is approved by the FDA as a reversal agent for overcoming MDR mediated by ABC transporters. It would be important to find effective drugs to overcome MDR mediated by these transporters (21).

**Figure 1:**
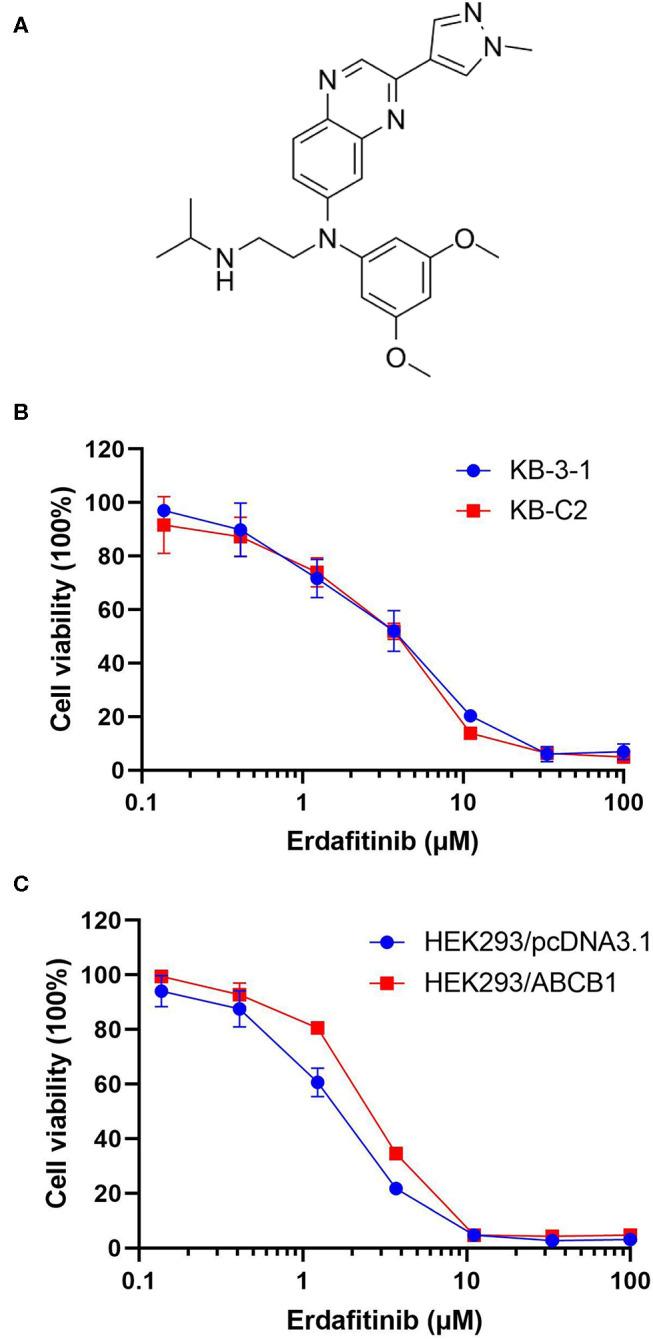
The cytotoxicity of erdafitinib in different cells. **(A)** The chemical structure of erdafitinib. **(B)** The cell viability curve of KB-C2 and KB-3-1 cells. **(C)** The cell viability curve of HEK293/ABCB1 and HEK293/pcDNA3.1 cells. The results are representative of three independent experiments. Error bars indicate SD.

In recent years, some tyrosine kinase inhibitors (TKIs), including dasatinib, glesatinib, and imatinib, have been demonstrated to exhibit MDR reversal effect (15, 22). Although there is no drug approved by FDA for reversing MDR, using FDA-approved drug combination has become an alternative strategy to overcome MDR (8, 23). Erdafitinib (Figure 1A) is a TKI which affect the fibroblast growth factor receptor (FGFR) to inhibit the tyrosine kinase activities of FGFR1-4 (24). It is the first FGFR kinase inhibitor approved by the FDA in 2019 to treat urothelial carcinoma. Additionally, several clinical trials evaluating the effect of erdafitinib on hepatocellular carcinoma (NCT02421185), breast cancer (NCT03238196), non-small-cell lung cancer (NCT02699606), and prostate cancer (NCT03999515) have been initiated. The present study evaluates if erdafitinib could reverse MDR mediated by ABCB1 in drug resistant cancer cells.

## Materials and Methods

### Chemicals

Erdafitinib was kindly offered as a gift from ChemieTek (Indianapolis, IN). Fetal bovine serum (FBS) and Dulbecco's modified Eagle's Medium (DMEM) were purchased from Corning Incorporated (Corning, NY). The monoclonal antibodies against GAPDH and ABCB1 were bought from Thermo Fisher Inc. (Rockford, IL). MTT {3-(4,5-dimethylthiazolyl)-2,5-diphenyltetrazolium bromide}, Triton X-100, 4′,6-diamidino-2-phenylindole (DAPI), paraformaldehyde, vincristine, paclitaxel, cisplatin, verapamil, Ko143, and other reagents were purchased from Sigma-Aldrich (St. Louis, MO).

### Cell Culture

ABCB1-overexpressing cell line KB-C2 was selected by colchicine with human epidermoid carcinoma cell line KB-3-1 (25). ABCG2-overexpressing cell line NCI-H460/MX20 was selected by mitoxantrone with human lung cancer cell line NCI-H460 (26). HEK293/pcDNA3.1 and HEK293/ABCB1 were obtained by transfecting HEK293 cells with empty pcDNA3.1 or ABCB1 expressing vector (27). All cell lines were cultured in DMEM supplemented with 10% FBS.

### Cytotoxicity and MDR Reversal Experiments

The cytotoxicity and MDR reversal experiments were performed by the MTT assay as previously reported (17). In brief, for erdaftinib cytotoxicity study, cells were seeded overnight in 96-well plates (5 × 10^3^ cells/well). Different concentrations of erdafitinib were added. For reversal study, different concentrations of paclitaxel, vincristine, or cisplatin were added into the wells after cells pre-incubated with different concentrations (0.06, 0.2, or 0.6 μM) of erdafitinib for 2 h. The cells were incubated for 72 h. Then 20 μL MTT (4 mg/mL) was added to each well for 4 h. After that, the medium with MTT was removed and then 100 μL DMSO was added to each well. The light absorbance values were measured using the accuSkanTM GO UV/Vis Microplate Spectrophotometer (Fisher Sci., Fair Lawn, NJ). Resistance fold (RF) was calculated by dividing the IC_50_ values of resistant cells by the IC_50_ of parental cells in the presence or absence of erdafitinib or positive control inhibitor. The known ABCB1 inhibitor, verapamil, was used as a positive control inhibitor. Cisplatin, a non-substrate drug of ABCB1, was used as a negative control anticancer drug for reversal study.

### Western Blot and Immunofluorescence Analysis on ABCB1 Expression

We carried out the Western blot analysis as described previously (28). In brief, cells were treated with 0.6 μM of erdafitinib for 0, 24, 48, or 72 h at 37°C, and lysed. Equal amounts (10 μg) of proteins were subjected to SDS-PAGE and then transferred to PVDF membranes. The membranes were incubated with anti-ABCB1 (1:1000) and anti-GAPDH (1:1000) overnight at 4°C. After incubating with secondary antibody (1:2000), the bands were analyzed using the enhanced chemiluminescence reaction kit (Amersham, NJ).

Immunofluorescent staining was performed as reported previously (18). In brief, cells were seeded in 24-well plate, and then treated with 0.6 μM of erdafitinib for 0, 24, 48, or 72 h. Briefly, the cells were fixed using 4% formaldehyde for 15 min and then incubated with ABCB1 antibody (1:500) overnight. Subsequently, cells were incubated with Alexa Fluor 488 conjugated IgG secondary antibody (1:2000) and DAPI. The cells were visualized using a Nikon TE-2000S fluorescence microscope (Nikon Instruments Inc., Melville, NY) and photographs were taken.

### [^3^H]-Paclitaxel Accumulation and Efflux Assay

As described in the previous study (15), the drug resistant cells and parental cells were seeded into 24-well plates. Cells were cultured overnight to allow for attachment. Each well was pre-treated with or without a reversal agent erdafitinib or verapamil for 2 h. After that, cells were incubated in complete DMEM medium containing 5 nM of [^3^H]-paclitaxel with or without a reversal agent for another 2 h. After that, the cells were rinsed with PBS, trypsinized, harvested, and placed in liquid scintillation cocktail (VWR Chemicals, Solon, OH, USA), and then collected into scintillation vials. To further assess the efflux function mediated by ABCB1, the [^3^H-paclitaxel efflux assay was conducted based on established protocol (17). In short, cells were prepared in the same way as the accumulation assay. Then, cells were cultured with or without an inhibitor (erdafitinib or verapamil) at indicated concentrations at 37°C. Following 2 h incubation period, cells were cultured in the presence or absence of an inhibitor for serial time frames (0, 30, 60, and 120 min). Subsequently, cells were trypsinized, harvested, and transferred into liquid scintillation solution.

The radioactivity was read using the Packard TRICARB 1900CA liquid scintillation analyzer (Packard Instrument, Downers Grove, IL).

### ATPase Assay

The ATPase activity of ABCB1 upon erdafitinib (0–1 μM) treatment was measured by PREDEASY ATPase Kits (TEBU-BIO nv, Boechout, Belgium) according to manufacturer's instructions (29). In short, different concentrations of erdafitinib were incubated with ABCB1 over expressing cell membranes for 3 min, and then 5 mM Mg^2+^ ATP was added to start the ATPase reaction. After incubation at 37°C for 40 min, luminescence signals of Pi were measured. The changes of relative light units were determined by comparing Na_3_VO_4_-treated samples with erdafitinib-treated groups.

### Docking Analysis

Molecular docking on erdafitinib with human ABCB1 model was performed as previously described (30). Briefly, human ABCB1 protein model (6QEX) was obtained from RCSB Protein Data Bank. Docking calculation was performed in AutoDock Vina (version 1.1.2) (31). Receptor/ligand preparation and docking simulation were performed with default parameters.

### Statistical Analysis

The data were expressed as mean ± SD. Data was analyzed by ANOVA followed by the Dunnett's test. Curve plotting and statistical analysis were achieved using GraphPad Prism 8.00 (La Jolla, CA, USA). *P* < 0.05 represents statistical significance. All experiments were repeated at least three times.

## Results

### Erdafitinib Increased the Sensitivity of Chemotherapeutic Drugs in ABCB1-Overexpressing Cells

The cytotoxicity of erdafitinib was determined by MTT assay. The non-cytotoxic concentrations (lower than IC_20_ values), 0.06, 0.2, and 0.6 μM of erdafitinib were selected and applied for the following experiments (Figures 1B,C).

The cytotoxicity of several ABCB1 substrate drugs, including vincristine and paclitaxel, with or without co-treatment with erdafitinib at 0.06, 0.2, and 0.6 μM was tested. As an inhibitor of ABCB1, verapamil was used as a positive control. Cisplatin, which is not a substrate drug of ABCB1, was used as a negative control. As shown in Table 1, ABCB1 overexpressing KB-C2 cells exhibited greater drug resistant fold compared to KB-3-1 cells, by 171.5- and 128.0-fold to paclitaxel and vincristine, respectively. Erdafitinib significantly sensitized the drug resistant cells to paclitaxel and vincristine in a concentration-dependent manner. More importantly, erdafitinib showed stronger sensitizing effect than verapamil at the same concentration. Erdafitinib (0.6 μM) could sensitize KB-C2 cells and the reversal effect was similar to that of verapamil at 3 μM. Meanwhile, the reversal effect of erdafitinib was assessed in ABCB1-transfected cells. As shown in Table 2, erdafitinib showed similar sensitizing effect to ABCB1-transfected HEK293/ABCB1 cells, and 0.6 μM of erdafitinib could completely reverse the drug resistance to paclitaxel and vincristine.

**Table 1:** Erdafitinib reversed ABCB1-mediated MDR in KB-C2 cells.

**Treatment**	**IC_****50****_**value±SD**^a^**(μM, Resistance fold**^b^)**	
	**KB-3-1**	**KB-C2**
Paclitaxel	0.031 ± 0.012 (1.0)	5.318 ± 0.514 (171.5)
**+** Erdafitinib 0.06 μM	0.036 ± 0.008 (1.2)	1.465 ± 0.125^*^ (47.3)
**+** Erdafitinib 0.2 μM	0.032 ± 0.015 (1.0)	0.774 ± 0.094^*^ (25.0)
**+** Erdafitinib 0.6 μM	0.038 ± 0.009 (1.2)	0.146 ± 0.007^*^ (4.7)
+Verapamil 0.6 μM	0.030 ± 0.009 (1.0)	0.938 ± 0.428^*^ (30.3)
+Verapamil 3 μM	0.028 ± 0.007 (0.9)	0.129 ± 0.026^*^ (4.2)
Vincristine	0.012 ± 0.004 (1.0)	1.536 ± 0.252 (128.0)
**+** Erdafitinib 0.06 μM	0.010 ± 0.003 (0.8)	0.597 ± 0.138^*^ (49.8)
**+** Erdafitinib 0.2 μM	0.012± 0.006 (1.0)	0.313 ± 0.102^*^ (26.1)
**+** Erdafitinib 0.6 μM	0.013 ± 0.003 (1.1)	0.042 ± 0.002^*^ (3.5)
+Verapamil 0.6 μM	0.010 ± 0.002 (0.8)	0.469 ± 0.089^*^ (39.1)
+Verapamil 3 μM	0.011 ± 0.004 (0.9)	0.034 ± 0.003^*^ (2.8)
Cisplatin	2.689 ± 0.314 (1.0)	2.818 ± 0.358 (1.0)
**+** Erdafitinib 0.06 μM	3.068 ± 0.380 (1.1)	3.001± 0.374 (1.1)
**+** Erdafitinib 0.2 μM	2.734 ± 0.166 (1.0)	2.889 ± 0.374 (1.1)
**+** Erdafitinib 0.6 μM	3.257 ± 0.473 (1.2)	2.890 ± 0.051 (1.1)
+Verapamil 0.6 μM	2.785 ± 0.294 (1.0)	3.013 ± 0.227 (1.1)
+Verapamil 3 μM	2.635 ± 0.162 (1.0)	3.048 ± 0.530 (1.1)

a *IC_50_ values are represented as mean ± SD of three independent experiments that were performed in triplicate*.

b *Rf: Resistance fold was calculated by dividing the IC_50_ values of substrates in the presence or absence of inhibitor by the IC_50_ of parental cells without inhibitor*.

* *P < 0.05 vs. the control*.

**Table 2:** Erdafitinib reversed ABCB1-mediated MDR in HEK293/ABCB1 cells.

**Treatment**	**IC_****50****_**value±SD**^a^**(μM, Resistance fold**^b^)**	
	**HEK293/pcDNA3.1**	**HEK293/ABCB1**
Paclitaxel	0.021 ± 0.003 (1.0)	0.881 ± 0.038 (42.0)
**+** Erdafitinib 0.06 μM	0.019± 0.007 (0.9)	0.287 ± 0.062^*^ (13.7)
**+** Erdafitinib 0.2 μM	0.015 ± 0.005 (0.7)	0.104 ± 0.056^*^ (5.0)
**+** Erdafitinib 0.6 μM	0.019 ± 0.006 (0.9)	0.028 ± 0.007^*^ (1.3)
+Verapamil 0.6 μM	0.015 ± 0.005 (0.7)	0.264 ± 0.066^*^ (12.6)
+Verapamil 3 μM	0.023 ± 0.001 (1.1)	0.030 ± 0.012^*^ (1.4)
Vincristine	0.005 ± 0.002 (1.0)	0.297 ± 0.040 (59.4)
**+** Erdafitinib 0.06 μM	0.006 ± 0.003 (1.2)	0.110 ± 0.034^*^ (22.0)
**+** Erdafitinib 0.2 μM	0.004 ± 0.001 (0.8)	0.009 ± 0.003^*^ (1.8)
**+** Erdafitinib 0.6 μM	0.005 ± 0.003 (1.0)	0.006 ± 0.003^*^ (1.2)
+Verapamil 0.6 μM	0.004 ± 0.002 (0.8)	0.192 ± 0.047^*^ (38.4)
+Verapamil 3 μM	0.004 ± 0.002 (0.8)	0.016 ± 0.006^*^ (3.2)
Cisplatin	2.809 ± 0.334 (1.0)	2.780 ± 0.453 (1.0)
**+** Erdafitinib 0.06 μM	2.927 ± 0.072 (1.0)	3.535 ± 0.410 (1.3)
**+** Erdafitinib 0.2 μM	3.110 ± 0.080 (1.1)	3.421 ± 0.260 (1.2)
**+** Erdafitinib 0.6 μM	3.174 ± 0.219 (1.1)	3.353 ± 0.341 (1.2)
+Verapamil 0.6 μM	2.991 ± 0.075 (1.1)	3.202 ± 0.178 (1.1)
+Verapamil 3 μM	2.627 ± 0.091 (0.9)	2.902 ± 0.796 (1.0)

a *IC_50_ values are represented as mean ± SD of three independent experiments that were performed in triplicate*.

b *Rf: Resistance fold was calculated by dividing the IC_50_ values of substrates in the presence or absence of inhibitor by the IC_50_ of parental cells without inhibitor*.

* *P < 0.05 vs. the control*.

In addition, we also evaluated if erdafitinib could reverse ABCG2-mediated MDR. Mitoxantrone is a substrate of ABCG2 and was used as a positive control. Cisplatin is not a substrate of ABCG2 and was used as a negative control. As shown in Table 3, erdafitinib failed to reverse MDR mediated by ABCG2 in NCI-H460/MX20 cells. This result suggested that erdafitinib specifically reverses MDR mediated by ABCB1.

**Table 3:** Erdafitinib did not reverse ABCG2-mediated MDR.

**Treatment**	**IC_****50****_**value±SD**^a^**(μM, Resistance fold**^b^)**	
	**NCI-H460**	**NCI-H460/MX20**
Mitoxantrone	0.048 ± 0.010 (1.0)	4.252 ± 0.606 (88.6)
**+** Erdafitinib 0.06 μM	0.027 ± 0.006 (0.6)	3.645 ± 1.063 (75.9)
**+** Erdafitinib 0.2 μM	0.033 ± 0.006 (0.7)	4.089 ± 0.873 (85.2)
**+** Erdafitinib 0.6 μM	0.047 ± 0.029 (1.0)	3.697 ± 1.062 (77.0)
+Ko143 3 μM	0.046 ± 0.015 (1.0)	0.273 ± 0.028^*^ (5.7)
Cisplatin	1.868 ± 0.243 (1.0)	2.004 ± 0.149 (1.1)
**+** Erdafitinib 0.06 μM	1.667 ± 0.230 (0.9)	1.836 ± 0.465 (1.0)
**+** Erdafitinib 0.2 μM	2.050 ± 0.385 (1.1)	2.093 ± 0.203 (1.1)
**+** Erdafitinib 0.6 μM	1.973 ± 0.170 (1.1)	1.765 ± 0.110 (0.9)
+Ko143 3 μM	1.646 ± 0.105 (0.9)	2.276 ± 0.152 (1.2)

a *IC_50_ values are represented as mean ± SD of three independent experiments which were performed in triplicate*.

b *Rf: Resistance fold was calculated by dividing the IC_50_ values of substrates in the presence or absence of inhibitor by the IC_50_ of parental cells without inhibitor*.

* *P < 0.05 vs. the control*.

### Erdafitinib Did Not Modify the Expression of ABCB1 or Its Cellular Localization

**Figure 2:**
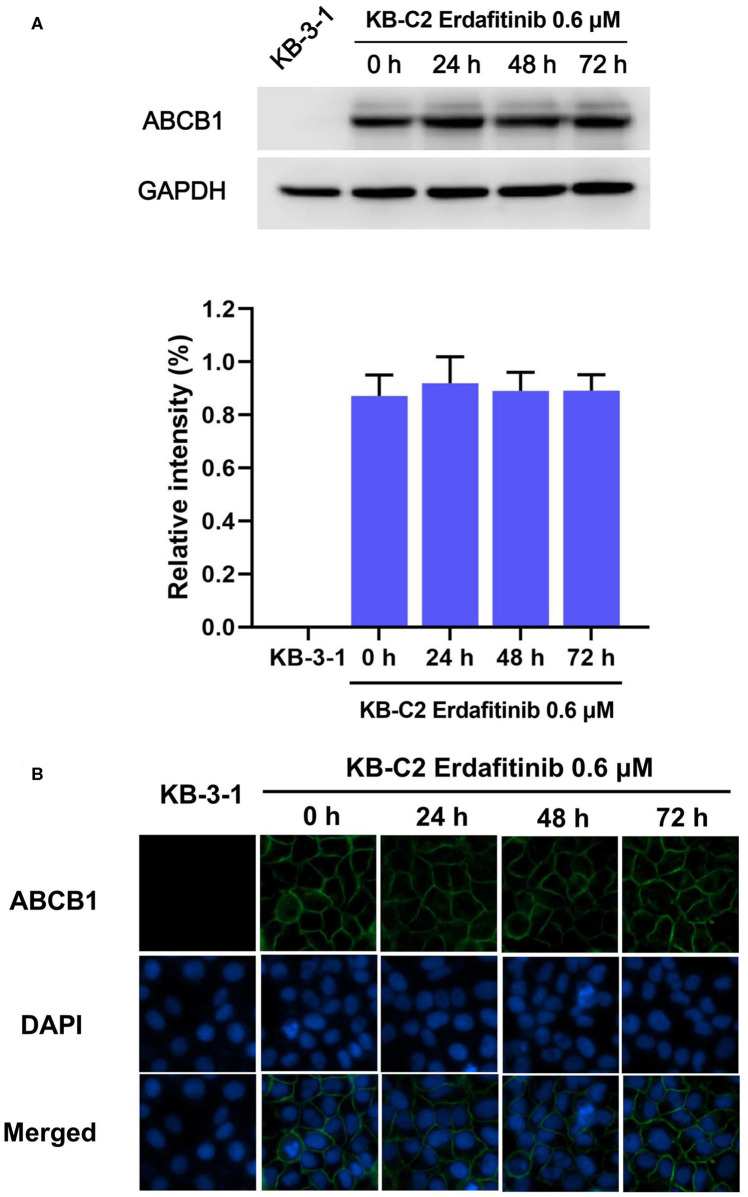
Erdafitinib modified neither the expression nor the cellular localization of ABCB1 in KB-C2 cells. **(A)** The effect of erdafitinib on ABCB1 expression in KB-C2 cells. **(B)** The effect of erdafitinib on the cellular localization of ABCB1 in KB-C2 cells. The results are representative of three independent experiments. Error bars indicate SD. Green: ABCB1. Blue: nuclei.

Previous studies reported that the mechanisms of reversal effect included down-regulating expression or altering cellular localization of ABCB1 and inhibition of the ABCB1 efflux activity. In order to explore the potential mechanisms, Western blot and immunofluorescence were carried out. Western blot analysis showed that at 0.6 μM, erdafitinib did not change the ABCB1 expression after 72 h treatment period (Figure 2A). Also, the immunofluorescence staining showed that at 0.6 μM, erdafitinib did not change the cellular localization of ABCB1 transporter (Figure 2B). These results revealed that erdafitinib modified neither the expression nor the localization of ABCB1.

### Erdafitinib Increased the Intracellular [^3^H]-Paclitaxel Accumulation and Inhibited [^3^H]-Paclitaxel Efflux in Cancer Cells Overexpressing ABCB1

**Figure 3:**
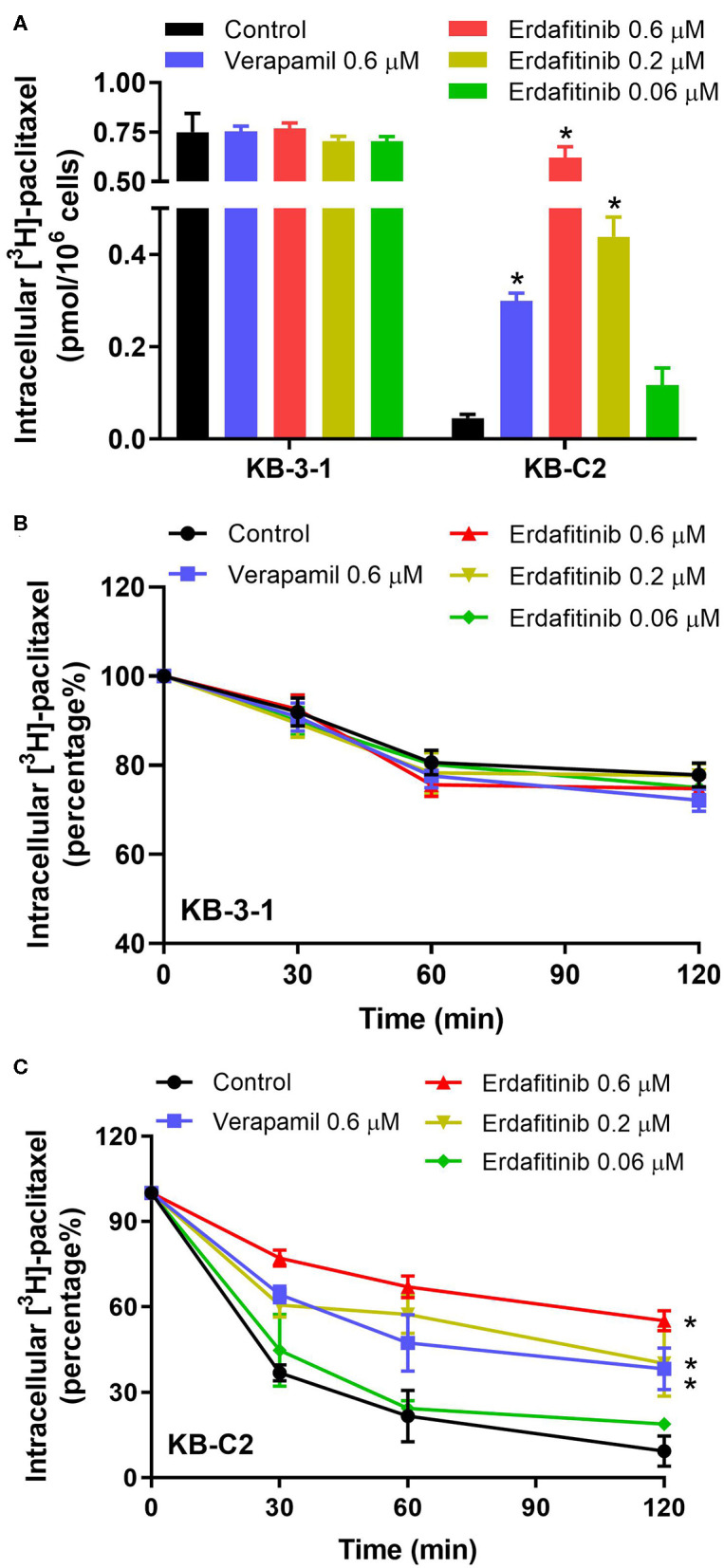
The effect of erdafitinib on the accumulation and efflux of [^3^H]-paclitaxel in KB-3-1 and KB-C2 cells. **(A)** The effect of erdafitinib on the accumulation of [^3^H]-paclitaxel in KB-3-1 and KB-C2 cells. **(B)** The effect of erdafitinib on the efflux of [^3^H]-paclitaxel in KB-3-1 cells. **(C)** The effect of erdafitinib on the efflux of [^3^H]-paclitaxel in KB-C2 cells. The results are representative of three independent experiments. Error bars indicate SD. *P < 0.05 vs. control. Positive control: the group of verapamil 0.6 μM.

Since the results mentioned above revealed that erdafitinib modified neither the expression nor the cellular localization of ABCB1, we expected that erdatinib might inhibit the function of ABCB1. To evaluate the ABCB1 activity, the intracellular concentration of tritium-labeled paclitaxel was quantified in parental and ABCB1 overexpressing cell lines with or without an inhibitor of ABCB1. By performing [^3^H]-paclitaxel accumulation assay, we observed that erdafitinib increased the paclitaxel accumulation in drug-resistant KB-C2 cells in a concentration-dependent manner with no change in drug-sensitive KB-3-1 cells (Figure 3A). Therefore, the reversal effect of erdafitinib, at least in part, can be attributed to increased paclitaxel accumulation. Subsequently, [^3^H]-paclitaxel efflux assay was performed to investigate whether erdafitinib enhances paclitaxel accumulation by increasing paclitaxel uptake and/or inhibiting paclitaxel efflux. As shown in Figures 3B,C, erdafitinib did not affect paclitaxel efflux in drug-sensitive KB-3-1 cells, which is consistent with the result of paclitaxel accumulation (Figure 3A). In drug-resistant KB-C2 cells, erdafitinib treatment significantly hindered paclitaxel efflux. Hence, this result showed that erdafitinib could inhibit the efflux activity of ABCB1 resulted in increasing paclitaxel accumulation.

### Erdafitinib Activated the ABCB1-Associated ATPase

**Figure 4:**
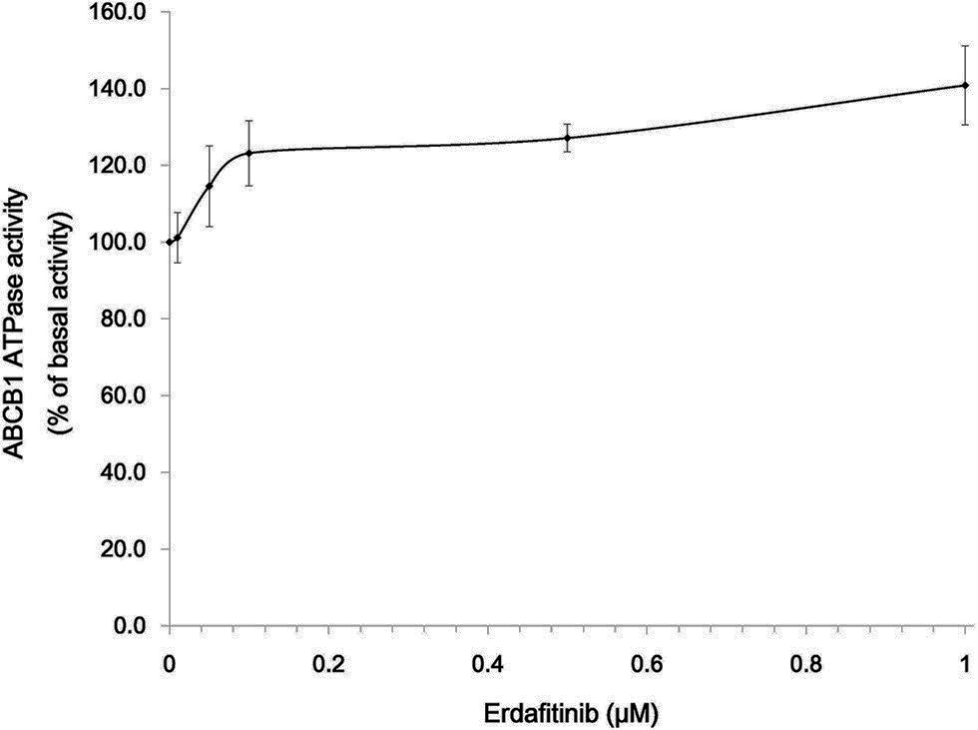
Erdafitinib increased the ATPase of ABCB1. Data are expressed as mean ± SD. The results are representative of three independent experiments. Error bars indicate SD.

It is reported that ATP hydrolysis is the energy source of ABC transporter to pump out endogenous and exogenous toxicants (32). Hence, we evaluated whether erdafitinib affects the ATPase activity of ABCB1. The vanadate-sensitive ABCB1-associated ATPase activity at different concentrations of erdafitinib (0–1 μM) was measured. As shown in Figure 4, erdafitinib concentration-dependently activated the ABCB1-associated ATPase to a maximum of 140.8% of the basal activity. The stimulatory effect of erdafitinib reached 50% maximal (EC_50_) at 0.07 μM.

### Docking Simulation of Erdafitinib in Human ABCB1

**Figure 5:**
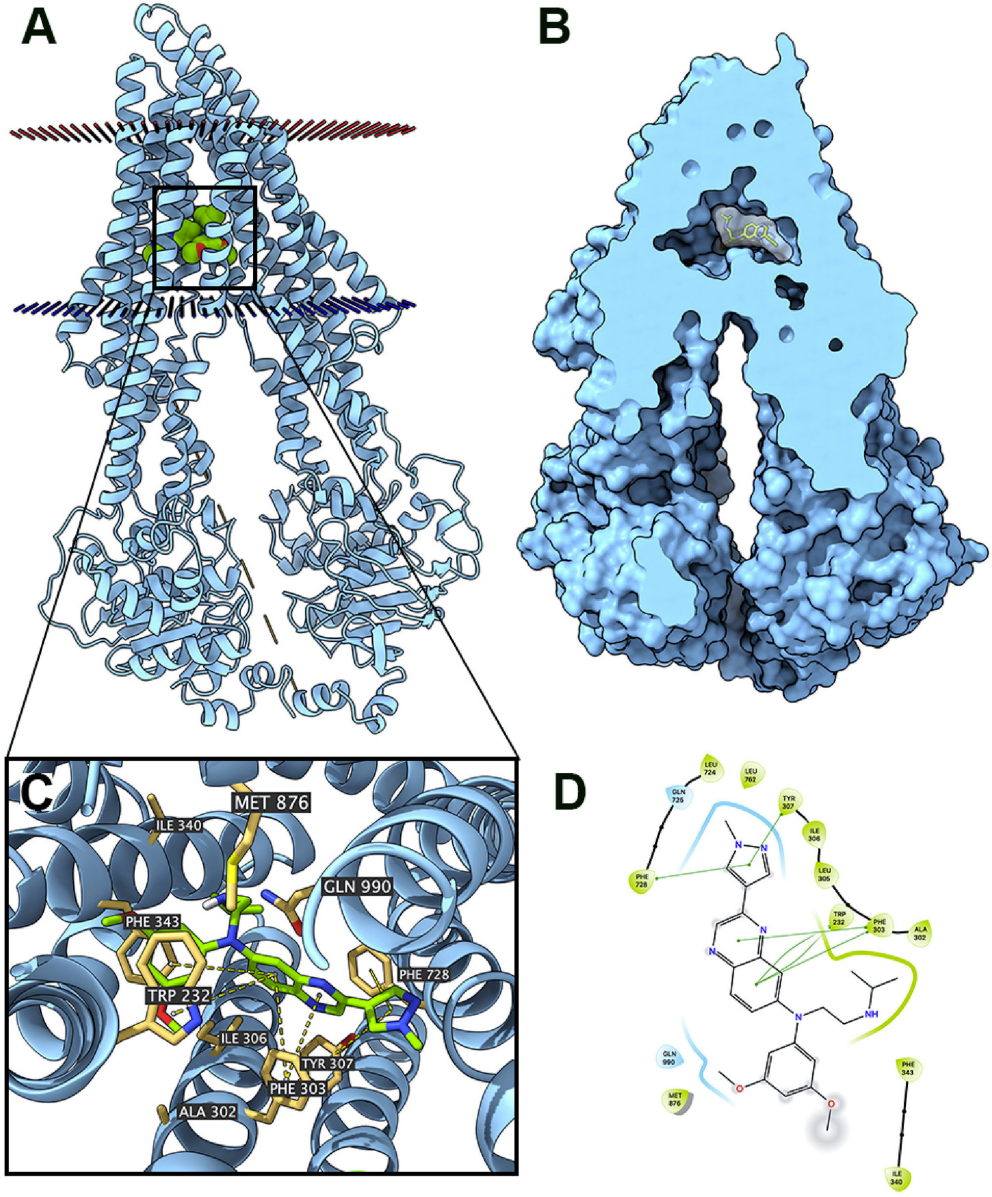
Interaction between erdafitinib and human ABCB1 protein. **(A)** Overview of the best-scoring pose of erdafitinib in the drug binding pocket of ABCB1 protein. Cytoplasmic membrane was depicted as dotted planes where red or blue plane indicate extracellular or intracellular side, respectively. ABCB1 was displayed as blue ribbons. Erdafitinib was displayed as colored spheres. Green: carbon; red: oxygen; blue: nitrogen. **(B)** Docked complex displayed with protein surface and ligand surface. Erdafinitib was displayed as colored sticks with white surface. **(C)** Details of interactions between erdafitinib and ABCB1 binding pocket. ABCB1 was displayed as blue ribbons. Important residues were displayed as colored sticks (wheat yellow: carbon; yellow: sulfur; blue: nitrogen; red: oxygen). Erdafitinib was displayed as colored sticks (lime green: carbon; blue: nitrogen; red: oxygen). π-π stackings were displayed as yellow dash lines. **(D)** 2D erdafitinib-ABCB1 interaction. Amino acids with 4.0 Å were displayed as color bubbles, cyan indicates polar residues, and green indicates hydrophobic residues. π-π stacking interactions are indicated with green lines.

The interaction between erdafitinib and the human ABCB1 model was determined by docking simulation. Erdafitinib was docked into the ABCB1 drug-binding sites with a high affinity score of −8.5 kcal/mol. Details of the ligand-receptor interaction was displayed in Figure 5. According to the docked complex, hydrophobic interactions were the major factor in the binding of erdafitinib to ABCB1 protein. Specifically, the pyrazole ring of erdafitinib was stabilized by the phenyl rings of Tyr307 and Phe728 of ABCB1 through π-π stacking interactions. Similarly, the center quinoxaline of erdafitinib was also stabilized by π-π stacking interactions with the phenyl rings of Phe303 and Trp232. Additionally, erdafitinib is positioned in a hydrophobic cavity formed by Trp323, Phe303, Ala302, Phe343, and Ile340.

## Discussion

It was known that ABCB1 overexpression could cause MDR, leading to chemotherapy failure (3, 33, 34). It is important to search for agents that could overcome MDR mediated by ABCB1. However, no ABCB1 inhibitor has been approved by FDA owing to severely adverse effect of candidate drugs (35–37). Recently, many studies suggested that combinations of chemotherapeutic drugs and a TKI could reverse ABCB1-mediated MDR (38). Erdafitinib is an FGFR kinase inhibitor for urothelial carcinoma approved by the FDA. In this study, we evaluate whether erdafitinib could reverse ABCB1-mediated MDR.

We found that erdafitinib could significantly reverse MDR mediated by ABCB1. The reversal effect of erdafitinib was stronger than that of verapamil, which is a known ABCB1 inhibitor. It is worth noting that similar results were obtained in both drug selected ABCB1 overexpressing KB-C2 cells and HEK293/ABCB1 cells that were transfected with *ABCB1* gene. Therefore, our data suggested that erdafitinib specifically reversed MDR mediated by ABCB1.

There was reports that a few TKIs may conduct reversal effect by down regulation of ABCB1 expression or altering its cellular localization (39). Therefore, Western blot and immunofluorescence for ABCB1 with or without erdafitinib treatment were carried out to test these possibilities. It was found that erdafitinib changed neither the expression nor the localization of ABCB1. It should be noted that cells were treated by 0.6 μM of erdafitinib for 72 h in this study. Thus, further research is needed to test if erdafitinib with higher concentrations or longer incubation time could affect ABCB1 expression or cellular localization.

As the reversal effect of erdafitinib may be attributed to several mechanisms, [^3^H]-paclitaxel accumulation assay was performed to investigate if erdafitinib could enhance the paclitaxel accumulation level. The result suggested that the intracellular level of paclitaxel increased in drug-resistant KB-C2 cells with no change in parental KB-3-1 cells in the presence of erdafitinib. Since ABCB1 transporter is the major factor that confers resistance to paclitaxel in KB-C2 cells, erdafitinib may interact with ABCB1 to increase accumulation of ABCB1 substrate drug, such as paclitaxel. There are two possibilities by which erdafitinib can enhance ABCB1 substrate accumulation, either increase substrate uptake or decrease substrate efflux. The [^3^H]-paclitaxel efflux assay was carried out to investigate if erdafitinib is able to inhibit the efflux of paclitaxel. It was found that erdafitinib could inhibit the efflux of paclitaxel in drug-resistant KB-C2 cells. Hence, erdafitinib could increase the intracellular accumulation of substrate drug by hindering the efflux function of ABCB1, thereby reversing MDR. Of note, the effect of erdafitinib was stronger than the known ABCB1 inhibitor verapamil, suggesting erdafitinib is a potent ABCB1 inhibitor.

ABCB1 pumps out xenobiotics by consuming ATP (40, 41). Therefore, ATPase assay of ABCB1 was performed to test if erdafitinib could alter the ATPase activity of ABCB1. Our results showed that erdafitinib concentration-dependently activated the ABCB1-associated ATPase, consisting with previous studies (42, 43). Some ABC transporter substrate drugs can act as competitive inhibitors that bind to a distinct substrate-binding site and inhibit the efflux of a particular class of substrates (44). We found that erdafitinib could stimulate the function of ATPase of ABCB1. It should be noted that, although several ABCB1 inhibitors were identified as stimulators of ATPase of ABCB1, ABCB1 overexpression might not necessarily cause drug resistance to those inhibitors (15, 45). Further study is needed to determine if erdafitinib is an ABCB1 competitive inhibitor. Furthermore, docking analysis was performed. We found that erdafitinib was docked into drug-binding pocket of ABCB1 with a high affinity, thereby inhibiting the binding of other substrate drugs of ABCB1. Our result was in consistent with other reports. For example, several TKIs, including glesatinib, TMP195, ulixertinib, and olmutinib, could bind to the drug-binding sites of ABC transporters, including ABCB1 and ABCG2, to reverse MDR (13, 15, 45, 46).

By performing the mechanistic studies, we found that erdafitinib can inhibit ABCB1 substrate efflux without affecting the ABCB1 transporter expression level or subcellular localization. At the same time, erdafitinib stimulated the ABCB1 ATPase activity, suggesting it can interact with the substrate-binding site of ABCB1 transporter. If erdafitinib binds to the substrate-binding site, it may act as a substrate to compete with other substrates for efflux pump. However, the cytotoxicity of erdafitinib showed no significant difference in parental and ABCB1-overexpressing cells, suggesting it may not be a good substrate. Our recent work showed that an inhibitor may cause a conformational change at the binding pocket and hinder the binding of other substrates (47). We suppose erdafitinib may act at the same way, stimulating a conformational change at the substrate-binding site to inhibit substrate efflux and reverse MDR. Future studies may focus on exploring the possibility of this mechanism of action.

In conclusion, our study demonstrated that as an anti-cancer drug approved by FDA, erdafitinib could antagonize ABCB1-mediated MDR. Combination of erdafitinib and a substrate of ABCB1 could improve the therapeutic effect for drug resistant cancers which overexpress ABCB1 transporter. This study result warrants evaluation in pre-clinical and clinical studies.

## Data Availability Statement

The raw data supporting the conclusions of this article will be made available by the authors, without undue reservation.

## Author Contributions

WF and D-HY designed the experiments. WF, MZ, Z-XW, J-QW, X-DD, and Q-XT performed the experiments. YY, X-YC, and QC analyzed the data. WF and MZ drafted the paper. D-HY edited the article. All authors read and approved the final manuscript. All authors contributed to the article and approved the submitted version.

## Conflict of Interest

The authors declare that the research was conducted in the absence of any commercial or financial relationships that could be construed as a potential conflict of interest.
